# Comparisons of school and home-packed lunches for fruit and vegetable dietary behaviours among school-aged youths

**DOI:** 10.1017/S136898001900017X

**Published:** 2019-02-26

**Authors:** Jennifer C Taylor, Carolyn Sutter, Lenna L Ontai, Adrienne Nishina, Sheri Zidenberg-Cherr

**Affiliations:** 1 The Design Lab, University of California, San Diego, 9500 Gilman Drive, MC 0436, La Jolla, CA 92093-0109, USA; 2 Department of Nutrition, University of California, Davis, Davis, CA, USA; 3 Center for Nutrition in Schools, University of California, Davis, Davis, CA, USA; 4 Family Resiliency Center, University of Illinois, Urbana–Champaign, Urbana, IL, USA; 5 Department of Human Ecology, University of California, Davis, Davis, CA, USA; 6 University of California Agriculture and Natural Resources, Davis, CA, USA

**Keywords:** Fruits and vegetables, School nutrition, Digital imaging, Packed lunch, Dietary behaviour

## Abstract

**Objective:**

School-based interventions and policies encourage youths to include and consume fruits and vegetables at lunchtime via school lunches, but limited research has examined how these behaviours compare when youths have home-packed lunches. The objective of the present study was to compare fruit and vegetable contents and consumption among students having school or home-packed lunches over the school week.

**Design:**

Participants were observed over five consecutive days at school lunchtime. Trained analysts estimated students’ lunchtime fruit and vegetable contents and consumption using digital imaging. Mixed models examined associations between fruit and vegetable dietary behaviours and lunch source (school *v*. home-packed), controlling for student gender, grade and school.

**Setting:**

Three elementary schools in northern California, USA.

**Participants:**

Fourth-, fifth- and sixth-grade students (*n*_children_ 315; *n*_observations_ 1421).

**Results:**

Students were significantly less likely to have and to consume fruits and vegetables (all *P*<0·05) when having home-packed lunches, compared with when having school lunches. Among those who did have or did consume these foods, having a home-packed lunch was associated with consuming significantly less fruit (*P*<0·05) but no differences for other dietary outcomes.

**Conclusions:**

The study adds to a growing body of literature indicating shortfalls in fruit and vegetable contents and consumption associated with having a home-packed lunch, relative to having a school lunch. Findings suggest that school-based interventions, particularly when targeting home-packed lunches, should focus on whether or not these foods are included and consumed, with less emphasis on quantities.

Increasing consumption of fruits and vegetables from an early age may improve health outcomes. For example, fruits and vegetables may displace low-nutrient, energy-dense foods in a child’s diet that are associated with increased risk of obesity^(^ ^1^ ^)^. Additionally, greater consumption of fruits and vegetables is associated with reduced risk of chronic diseases such as CVD^(^ ^2^ ^)^. Schools can play an instrumental role in addressing the low adherence to fruit and vegetable recommendations among US children^(^ ^3^ ^,^ ^4^ ^)^, since youths consume up to half of their energy intake at school^(^ ^5^ ^,^ ^6^ ^)^. The introduction of updated nutrition standards for the National School Lunch Program (NSLP) in 2012^(^ ^7^ ^)^ has been associated with improvements in these dietary behaviours at school among NSLP participants^(^ ^8^ ^)^. However, close to half of students bring foods from home in packed lunches, which are not subject to these federal standards^(^ ^9^ ^)^.

A growing body of research observing lunchtime dietary behaviours indicates that contents of home-packed lunches are often of low nutritional quality and fall short of federal nutrition guidance^(^ ^10^ ^–^ ^14^ ^)^. Recognizing these shortfalls associated with home-packed lunches has stimulated the development of interventions targeting these lunches and addressing the home environment^(^ ^15^ ^,^ ^16^ ^)^.

Few studies have directly compared school and home-packed lunches with respect to students’ fruit and vegetable behaviours. Studies to date have indicated that home-packed lunches less often contain any fruits and vegetables when compared with school lunches^(^ ^17^ ^,^ ^18^ ^)^. These studies addressed whether or not fruits and vegetables are included in the lunch, but the extent to which the quantities of contents differ by lunch source is unknown. Comparisons of what is ultimately consumed from these lunch sources are also needed. While students having school lunches are required to include at least one-half cup of fruits and vegetables, a large proportion of fruits and vegetables contained within a school lunch may go uneaten, making contents (i.e. what is served to, and/or selected by, the student) a weak proxy for consumption^(^ ^19^ ^,^ ^20^ ^)^. By contrast, parents report that their decisions to pack a lunch from home are driven by the desire to offer foods they know their child likes and will eat, potentially resulting in the consumption of most or all of any fruit and vegetable contents^(^ ^21^ ^)^. Together, these findings suggest that students having home-packed lunches may consume equal, if not more, fruits and vegetables than those having school lunches, despite on average containing fewer servings of them. Hur *et al*.^(^ ^22^ ^)^ found that elementary-school students having home-packed lunches consumed more fruits but less vegetables than students having school lunches. This finding for vegetable consumption contrasts with the pattern reported for lunch contents, underscoring the importance of understanding both what is included and what is ultimately eaten from these lunch sources.

Finally, it is important to consider the types of methods used to examine dietary behaviours. Self-reported dietary assessment methods have well-known limitations related to respondent burden and measurement error that can limit their feasibility and validity when working with school-aged youths^(^ ^23^ ^,^ ^24^ ^)^. These limitations may be mitigated by working with observation-based plate waste methods that capture information on foods included (contents) and eaten (consumption) within a given meal occasion, such as the lunch period. While these methods are increasingly applied to studies examining home-packed lunches, limited research has observed school and home-packed lunches simultaneously, and fewer studies have observed both what is included and what is actually consumed. In addition, studies typically rely on a single lunch observation to measure dietary behaviour, but children’s and adolescents’ dietary behaviours show a high degree of day-to-day variability^(^ ^25^ ^,^ ^26^ ^)^. Measuring behaviours over multiple days may capture more reliable estimates of differences between school and home-packed lunches.

The objective of the present study was to compare students’ fruit and vegetable dietary behaviours across the school week when having a school *v*. a home-packed lunch.

## Methods

### Participants

Participants were 315 fourth-, fifth- and sixth-grade students participating in the Parents to Peers study, a project examining social and contextual correlates of students’ dietary behaviours at lunchtime^(^ ^27^ ^)^. This sample size was determined based on a primary study aim regarding peer interactions at lunchtime. Students were recruited from three elementary schools within one California school district, with schools selected on the basis of having varied proportions of students eligible for free or reduced-price meals through the NSLP (school A=34 %; school B=76 %; school C=53 %). Of the 541 students eligible to participate, parental consent and student assent was obtained for 321 students (68 % of consent forms returned, 87 % of returned forms provided consent). Participation rates varied across schools based on percentage of consent forms returned (61, 82 and 66 % from schools A, B and C, respectively) and the percentage of these forms providing consent (86, 79 and 94 % from schools A, B and C respectively). Dietary data were available for 319 students. Because the study focused on students having either a school or a home-packed lunch on a given day, observations where students chose both lunch types were excluded from analyses, resulting in a final sample of 315 students.

### Study design

Recruitment and data collection occurred from January to April 2016. Information packets including consent forms were distributed to students through each classroom, on average 2·5 weeks prior to each 1-week period of data collection. A $US 5 incentive was provided for returning a signed parent consent form (regardless of whether the parent provided consent or declined to participate). Students were compensated for their participation ($US 1 to the student and $US 1 to the student’s classroom per day). Using a micro-longitudinal study design, data were collected from each participant over a period of five consecutive school days (i.e. a 1-week period), such that lunch source could vary from day to day for a student. All participants in a given grade completed the study during the same week, with data collection completed over seven weeks (thirty-five days). Parents completed demographic surveys, and lunchtime observations using digital imaging were conducted each day of the study to determine lunch source and dietary intake. The study was approved by the University of California, Davis, Institutional Review Board.

### Procedure

Fruit and vegetable contents and consumption were determined daily for five days for each student using digital imaging procedures adapted from previous studies^(^ ^12^ ^,^ ^28^ ^)^. Protocols applied in the present study, including feasibility and reliability testing, are described in more detail elsewhere^(^ ^29^ ^)^. Briefly, digital images of lunch contents were collected prior to lunch (for home-packed lunches) or at the start of the lunch period as students exited the lunch line (for school lunches). Digital images of plate waste remaining were collected at the end of lunch, including food remains and empty packaging, for both school and home-packed lunches. Students were instructed to wait to dispose of any food or packaging until after plate waste imaging, and field observation notes were collected during lunch to account for instances where foods were gained or removed (e.g. early disposal or food sharing). Digital images were collected of lunch contents while positioned in a school lunch tray and/or on a mat board marked with one-inch gridlines. In addition to digital images, supplemental information on food preparation was gathered for school lunches (menu data collected from school nutrition services) and home-packed lunches (written descriptions collected by trained researchers).

Following data collection, trained researchers with established inter-rater reliability determined food types and quantities from written and image records using visual estimation methods drawn from prior studies^(^ ^28^ ^–^ ^30^ ^)^. Foods were first identified from digital images of lunch contents, using supplemental information to aid food identification as needed. For example, written descriptions of packed lunch contents were collected for mixed dish items such as sandwiches to aid in coding its individual components. The quantity included for each of these items in the lunch was then determined by using manufacturer labels (pre-packaged items), school menu information (for pre-portioned items) or visual estimation procedures (all other items). Foods were visually estimated by either: (i) determining the item’s dimensions, using the one-inch gridlines of the mat board in the image (e.g. diameter of an apple); or (ii) using a series of over 2000 reference images that displayed foods in varied portion sizes (e.g. images of baby carrots displaying portions in 10 g increments). Finally, food consumption was estimated by comparing images of initial lunch contents with images of plate waste to determine the percentage consumed for each item. Food descriptions and quantities were entered into the Nutrition Data System for Research (NDSR) software version 2014, developed by the Nutrition Coordinating Center (NCC), University of Minnesota, Minneapolis, MN, USA^(^ ^31^ ^)^. Fruit and vegetable outcomes were each summed across all items in a lunch. Fruit outcomes excluded fruit juice given its lower nutrient density, particularly dietary fibre, compared with whole fruits, and given the emphasis on increasing whole fruits within national dietary guidance^(^ ^32^ ^)^. Vegetable outcomes excluded fried potatoes given the high energy density relative to other vegetable sub-categories. Quantities were reported in half-cup servings based on the NCC Food Group Serving Count System.

### Analyses

Descriptive statistics were used to describe student demographics, lunch source patterns across the school week and prevalence of each dietary behaviour (fruit contents included, fruit consumption, vegetable contents included, vegetable consumption) across the total sample of observations. Mixed models were used to examine dietary behaviours as a function of daily lunch source (school lunch, home-packed lunch), incorporating a random intercept to account for nesting of observations within students. Dietary behaviour distributions were zero-inflated with positive skewness. For example, this distribution for fruit consumption indicated that many students did not consume any fruit on a given day of the week, and that among those who did consume fruit, most consumed smaller quantities (i.e. up to 1 half-cup serving) while far fewer students consumed larger quantities (i.e. greater than 3 half-cup servings). Given this pattern across all four dietary outcomes, each dietary outcome was examined in two models: (i) a binary component examining whether or not the behaviour occurred (e.g. fruit quantity included >0 half-cup servings; fruit quantity included =0 half-cup servings); and (ii) a continuous component examining quantities, when the behaviour did occur (e.g. quantity of fruit included, given that quantity included >0 half-cup servings). This approach was chosen to address not only how much was included and consumed of these foods across lunch sources (the continuous outcome), but also to describe the extent to which these dietary behaviours are initiated at all (the binary component).

Binary outcomes were examined using logistic mixed models. Log-odds were transformed to probabilities and odds ratios to estimate the probability of each dietary behaviour occurring and to compare the likelihood for home-packed lunches, relative to school lunches (reference group). Continuous outcomes were examined using linear mixed models. A series of data transformations were examined, and square-root transformations were selected on the basis of Shapiro–Wilks normality tests (*W*>0·95) and inspection of residual plots for each model. The fixed-effects parameter estimates and confidence intervals for these continuous outcomes are presented as back-transformed least-squares means, using a Tukey adjustment for multiple comparisons. Based on comparisons of model fit (assessed by log-likelihood and Akaike information criterion), all models were adjusted for student gender, grade and school. Analyses were conducted in the statistical software package SAS version 9.4 using PROC NLMIXED and PROC MIXED.

## Results

Sample characteristics are summarized in Table 1. A total of 1421 observations were collected from 315 students. Approximately half of these observations were of school lunches (53 %). Aggregated across the week, most students consistently had a school lunch every day (45 %) or consistently had a home-packed lunch (37 %), while 18 % of students varied day-to-day in lunch source. These patterns for lunch source were associated with school ( 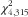 =107·0; *P*<0·0001), where most school A students consistently had a packed lunch (60 %), most school B students consistently had a school lunch (93 %) and school C students varied (i.e. 37 % consistently had a school lunch, 41 % consistently had a packed lunch and 22 % varied in lunch source).

**Table 1:** Characteristics of participants in a study examining dietary behaviours associated with lunch source among elementary-school students (*n*_children_ 315)†, northern California, USA, January–April 2016

	*n*	%
Gender		
Boy	140	44
Girl	175	56
Grade		
4	106	34
5	99	31
6	110	35
School		
A	88	28
B	75	24
C	152	48
Race/ethnicity		
African American/Black	27	9
Asian/Pacific Islander	21	7
Caucasian/White	112	37
Latino	55	18
Multi-ethnic	78	26
Other	10	3
Household income		
<$US 20 000	47	16
$US 20 000–39 999	57	20
$US 40 000–59 999	54	19
$US 60 000–79 999	31	11
$US 80 000–99 999	25	9
≥$US 100 0000	76	26

† Sample sizes differ due to missing data.

### Fruit contents

The probability of including fruit was 0·97 and 0·86 when having a school or a home-packed lunch, respectively. When having a home-packed lunch, students were 81 % less likely to include fruit than when having school lunches (OR=0·19 (95 % CI 0·11, 0·35), *P*<0·001; Table 2). Fruit quantities were examined after excluding two extreme observations where fruit contents exceeded 8 servings. On days when fruit was included (90 % of school and 72 % of home-packed lunch observations), the quantities included were not significantly different between school and home-packed lunches; lunches contained an average of 1·1 half-cup servings of fruit (Table 3).

**Table 2:** Associations between lunch source and odds of including and consuming fruits and vegetables among elementary-school students (*n*_children_ 315) observed over five school days (*n*_observations_ 1421), northern California, USA, January–April 2016

	Fruit contents† (*n*_children_ 315, *n*_observations_ 1421)		Fruit consumption (*n*_children_ 309, *n*_observations_ 1221)		Vegetable contents (*n*_children_ 315, *n*_observations_ 1421)		Vegetable consumption (*n*_children_ 312, *n*_observations_ 1300)	
	OR	95 % CI	OR	95 % CI	OR	95 % CI	OR	95 % CI
Lunch source								
School lunch	1·00	Reference	1·00	Reference	1·00	Reference	1·00	Reference
Packed lunch	0·19***	0·11, 0·35	0·31***	0·18, 0·52	0·60*	0·40, 0·92	0·56**	0·37, 0·86
Gender								
Boy	1·00	Reference	1·00	Reference	1·00	Reference	1·00	Reference
Girl	1·43	0·82, 2·49	1·40	0·84, 2·31	1·44	0·95, 2·18	1·46	0·96, 2·22
Grade								
4	1·00	Reference	1·00	Reference	1·00	Reference	1·00	Reference
5	0·96	0·48, 1·92	0·97	0·52, 1·79	1·91*	1·14, 3·18	1·65	0·99, 2·75
6	0·73	0·37, 1·41	1·01	0·55, 1·83	0·92	0·56, 1·52	0·98	0·59, 1·61
School								
A	1·00	Reference	1·00	Reference	1·00	Reference	1·00	Reference
B	0·56	0·23, 1·40	0·44*	0·20, 0·98	1·05	0·56, 1·99	0·64	0·33, 1·22
C	0·52	0·27, 1·01	0·44**	0·24, 0·81	0·91	0·56, 1·50	0·78	0·47, 1·28

*P<0·05, **P<0·01, ***P<0·001.

† Sample sizes differ due to missing data.

**Table 3:** Associations between lunch source and quantities of fruit and vegetable contents and consumption (half-cup servings) among (*n*_children_ 315) observed over five school days (*n*_observations_ 1421), northern California, USA, January–April 2016†

	Fruit contents‡ (*n*_children_ 297, *n*_observations_ 1156)		Fruit consumption (*n*_children_ 264, *n*_observations_ 804)		Vegetable contents (*n*_children_ 260, *n*_observations_ 707)		Vegetable consumption (*n*_children_ 241, *n*_observations_ 572)	
Characteristic	Mean	95 % CI	Mean	95 % CI	Mean	95 % CI	Mean	95 % CI
Lunch source								
School lunch	1·11	1·04, 1·17	0·65^a^	0·59, 0·71	0·89	0·81, 0·98	0·58	0·51, 0·65
Packed lunch	1·17	1·09, 1·26	0·86^b^	0·78, 0·95	0·77	0·68, 0·87	0·49	0·42, 0·58
Gender								
Boy	1·13	1·06, 1·21	0·77	0·69, 0·84	0·82	0·73, 0·92	0·55	0·47, 0·63
Girl	1·15	1·09, 1·22	0·74	0·68, 0·80	0·84	0·77, 0·92	0·52	0·46, 0·59
Grade								
4	1·14^a^	1·06, 1·22	0·77	0·69, 0·85	0·80	0·71, 0·90	0·56	0·48, 0·65
5	1·08^a^	0·99, 1·17	0·72	0·64, 0·81	0·82	0·72, 0·92	0·48	0·40, 0·56
6	1·20^b^	1·12, 1·29	0·77	0·69, 0·85	0·87	0·77, 0·98	0·57	0·49, 0·67
School								
A	1·14^a,b^	1·05, 1·24	0·74^a,b^	0·65, 0·83	0·88	0·77, 1·00	0·64^a^	0·54, 0·75
B	1·28^a^	1·17, 1·39	0·87^a^	0·76, 0·98	0·88	0·75, 1·01	0·45^b^	0·36, 0·56
C	1·01^b^	0·95, 1·08	0·66^b^	0·60, 0·73	0·74	0·66, 0·82	0·53^a,b^	0·46, 0·60

^a,b^For each characteristic separately, mean values within a column with unlike superscript letters were significantly different (*P*<0·05).

† Fruit and vegetable outcomes represent instances where students included (contents) or ate (consumption) non-zero quantities (in half-cup servings), representing sub-samples of the full 1421 observations among 315 students.

‡ Estimates are presented as least-squares means.

### Fruit consumption

The probability of consuming fruit was 0·87 when having a school lunch, compared with 0·68 when having a home-packed lunch. Students were 69 % less likely to consume fruit when having a home-packed lunch compared with when having a school lunch (OR=0·31 (95 % CI 0·18, 0·52), *P*<0·001; Table 2). On days when fruit was consumed (73 % of school and 58 % of home-packed lunch observations), the quantities consumed were significantly greater among home-packed lunches (mean=0·86 (95 % CI 0·78, 0·95) half-cup servings) than school lunches (mean=0·65 (95 % CI 0·58, 0·71) half-cup servings, *P*<0·001; Table 3).

### Vegetable contents

The probability of including vegetables was 0·47 and 0·35 when having a school lunch or a home-packed lunch, respectively. Students were 40 % less likely to include vegetables when having a home-packed lunch compared with when having a school lunch (OR=0·60 (95 % CI 0·40, 0·92), *P*<0·05; Table 2). On days when vegetables were included (54 % of school and 45 % of home-packed lunch observations), the quantities included were marginally less among home-packed lunches (mean*=*0·77 (95 % CI 0·68, 0·87) half-cup servings) than among school lunches (mean=0·89 (95 % CI 0·81, 0·98) half-cup servings, *P*=0·06; Table 3).

### Vegetable consumption

The probability of consuming vegetables was 0·45 when having a school lunch, compared with 0·31 when having a home-packed lunch. Students were 44 % less likely to consume vegetables when having a home-packed lunch compared with when having a school lunch (OR=0·56 (95 % CI 0·37, 0·86), *P*<0·01; Table 2). On days when vegetables were consumed (47 % of school and 41 % of home-packed lunch observations), the quantities consumed were not significantly different between lunch sources, averaging approximately 0·5 half-cup servings of vegetables (Table 3).

## Discussion

The present study investigated elementary-school students’ lunchtime dietary behaviours in relation to lunch source. Building on prior research using observation-based studies and primarily addressing fruit and vegetable contents included in the lunch, the study compared contents as well as consumption between school and home-packed lunches. Understanding differences in fruit and vegetable dietary behaviours associated with lunch source may be informative to the design of school-based interventions targeting the lunchroom.

Fruit contents findings align with prior research in that the likelihood of including fruit was lower when having a home-packed lunch, compared with having a school lunch. However, it is important to note that fruit was frequently included in both lunch sources (90 and 72 % of school and home-packed lunch observations, respectively). Updated nutrition standards for the NSLP likely contribute to the high probability of including fruit among school lunches because fruit selection has increased in response to these standards requiring students to select at least one-half cup of fruits or vegetables^(^ ^20^ ^,^ ^33^ ^–^ ^35^ ^)^. When fruits were included in the lunch, students’ lunches contained an average of 1·1 half-cup servings (i.e. 0·55 cups) and quantities of fruit included did not differ by lunch source. In other words, when students’ lunches did include fruits, quantities (on average) tended to meet NSLP nutrition standards for fruit^(^ ^7^ ^)^ and contributed 25–33 % of Dietary Guidelines for Americans’ recommendations for fruit for many youths within this age range^(^ ^32^ ^)^. Together, these findings suggest that interventions addressing lunchtime fruit contents are most needed to address whether or not fruit is included in the first place, particularly for home-packed lunches given the lower likelihood of these foods being included within this lunch source.

Fruit consumption was significantly less likely when students had home-packed lunches than when having school lunches, paralleling prior lunch comparisons addressing fruit contents^(^ ^17^ ^,^ ^18^ ^)^. However, when examining the average quantities of fruit consumed among those who did consume these foods, significantly greater quantities were consumed when students had home-packed lunches, consistent with prior research conducted prior to the implementation of updated NSLP nutrition standards^(^ ^22^ ^)^. Given that quantities of fruits included did not differ between school and home-packed lunches in the present study, greater consumption of fruits among home-packed lunches could reflect a tendency for home-packed lunches to include fruits that match student preferences, thus reducing food waste. This aligns with other plate waste research in finding a smaller proportion of fruits is wasted among home-packed lunches than school lunches^(^ ^10^ ^,^ ^19^ ^,^ ^20^ ^,^ ^36^ ^–^ ^39^ ^)^.

Students’ lunches were significantly less likely to include vegetables when having home-packed lunches. Prior research found that 13–17 % of home-packed and 29–61 % of school lunches included vegetables^(^ ^17^ ^,^ ^18^ ^)^, which is relatively low compared with the present study for home-packed (45 %) and school lunches (54 %). This may reflect age-related differences, as the present study represents an older age group (grades 4–6) than earlier studies (pre-kindergarten to grade 2), and diet quality for vegetable consumption may increase as children progress from early childhood into adolescence^(^ ^40^ ^)^. The variability in estimates for vegetable contents among school lunches also suggests there may be other contextual influences affecting this dietary behaviour from day to day or across different schools. For example, vegetable contents could vary day-to-day in relation to the variety of vegetables offered in salad bars or in entrée items^(^ ^41^ ^,^ ^42^ ^)^. When students had vegetables in their lunch, there was a marginally significant trend where greater quantities were included when having a school lunch than when having a home-packed lunch. While vegetables were included in only half of all observations, the average quantities included when present were close to 1 serving (0·89 servings or 0·45 cups) in a school lunch and about 15 % lower for home-packed lunches (0·77 servings or 0·39 cups). Given that a minority of students include any vegetables at lunchtime across both lunch sources, these findings suggest interventions should prioritize addressing whether or not any vegetables are included at lunchtime, as opposed to focusing on quantities included.

Students were significantly less likely to consume vegetables when having home-packed lunches, although the quantities of these foods consumed among vegetable consumers did not differ by lunch source. Finally, relative to fruit-related dietary behaviours, the probability of including and consuming vegetables was low, below 0·5, indicating students are more likely to omit than to include these foods on a given day. Consumption of vegetables from school lunches has varied widely in previous studies, which estimate the percentage of these foods consumed to range from 27 to 70 %^(^ ^19^ ^,^ ^20^ ^,^ ^36^ ^–^ ^39^ ^)^. Meanwhile, 70–77 % of vegetable contents are consumed among home-packed lunches^(^ ^10^ ^)^. While having a home-packed lunch was associated with lower likelihood of consuming vegetables, there is a need for further promotion of these foods among all students regardless of lunch source.

In the present study, all models were adjusted for student gender, grade and school based on improved model fit, and significant associations between lunch source and dietary outcomes remained even after accounting for these covariates. Holding lunch source and other student characteristics (grade, gender) constant, there were significant differences in dietary behaviours between schools. These differences were not systematic and may relate to NSLP participation (e.g. as a proxy for socio-economic status) or other school characteristics beyond the scope of the present study but explored in other research, such as time available to eat^(^ ^36^ ^,^ ^43^ ^,^ ^44^ ^)^ or student–teacher relationships at lunchtime^(^ ^45^ ^)^. Meanwhile, dietary behaviours generally did not differ by grade or by gender. Prior research documents a decline in fruit consumption during childhood and adolescence, while vegetable consumption exhibits a more stable or inconsistent pattern^(^ ^3^ ^,^ ^40^ ^,^ ^46^ ^)^. A similar decline in fruit consumption was not observed in the present study based on grade-level differences, although the age range covered in the study was relatively narrow. While studies have reported greater consumption of fruits and vegetables among girls than boys^(^ ^3^ ^,^ ^46^ ^)^, no gender differences were observed in the final models in the present study.

Strengths of the present study include its use of multiple days of observations to capture reliable estimates of dietary behaviours over the school week, as well as its use of an objective digital imaging method to minimize respondent burden and measurement error when assessing lunchtime diets. In addition, the study examined a student sample size within the range of prior lunch comparison studies^(^ ^10^ ^,^ ^12^ ^,^ ^22^ ^)^, while incorporating repeated observations to yield over 1400 observations. However, there were important limitations to the present study that should guide future research. First, we cannot rule out the potential for measurement error due to reactivity if students’ dietary behaviours change in response to the presence of researchers. Preliminary observations were conducted in each lunchroom ahead of the official observation periods to acclimatize students to the presence of researchers. Observation-based methods such as digital imaging are advantageous over self-report methods to mitigate several sources of measurement error (e.g. omissions or intrusions due to poor memory retention, misestimation of portion sizes)^(^ ^23^ ^)^. Nevertheless, further research on observation-based methods, particularly digital imaging, is needed to better understand whether, and to what degree, being observed may alter behaviour.

Second, while the present study’s sample was diverse in terms of race/ethnicity and socio-economic characteristics, it was restricted to three schools. While all schools participating in the NSLP follow the same federal nutrition standards, there may be differences in how each implements the programme. For example, all schools within the present study offered a variety of fruit and vegetable options daily as part of salad bars, which may increase contents and consumption of these foods in school lunches, relative to schools without salad bars^(^ ^47^ ^,^ ^48^ ^)^. Future research should consider how these food environment characteristics may shape dietary behaviours among school lunches, via school-to-school and day-to-day variation in fruit and vegetable availability within the school lunch menu. Similarly, variations in home fruit and vegetable availability should be examined in relation to home-packed lunches.

## Conclusion

Youths’ consumption of fruits and vegetables falls well below national recommendations and schools are opportune contexts for promoting these behaviours as part of obesity and chronic disease prevention strategies. While federal nutrition standards for the NSLP can target dietary behaviours among students having school lunches, many bring packed lunches from home, which are not subject to these standards. The present study found that students having home-packed lunches are less likely to include and to consume fruits and vegetables, although quantities included and consumed did not follow the same systematic pattern. Participation in the NSLP may encourage youths to establish healthy dietary behaviours during the school day and these findings aid in identifying areas for improving fruit and vegetable dietary behaviours, particularly among students having home-packed lunches. For instance, students having home-packed lunches were less likely to consume any vegetables, but when vegetables were consumed, the amount eaten did not differ between lunch sources. These findings suggest interventions tailored towards parents and youths preparing home-packed lunches should focus on encouraging more frequent inclusion of fruits and vegetables across the school week, while less emphasis may need to be placed on how much to pack. Encouraging more frequent inclusion, and ultimately consumption, of vegetables is particularly needed given that vegetables were included in only half of lunch observations. These combined findings are informative to designing nutrition interventions targeting dietary behaviours at school to establish and maintain healthy dietary behaviours from an early age.
